# “läuft.” - a school-based multi-component program to establish a physically active lifestyle in adolescence: study protocol for a cluster-randomized controlled trial

**DOI:** 10.1186/1745-6215-14-416

**Published:** 2013-12-05

**Authors:** Vivien Suchert, Barbara Isensee, Julia Hansen, Maike Johannsen, Claus Krieger, Katrin Müller, Ingeborg Sauer, Burkhard Weisser, James D Sargent, Reiner Hanewinkel

**Affiliations:** 1 Institute for Therapy and Health Research, IFT-Nord, Harmsstrasse 2, Kiel 24114, Germany; 2 University of Hamburg School of Education, Psychology and Human Movement, Hamburg, Germany; 3 Christian-Albrechts-University of Kiel, Institute for Sports Science, Kiel, Germany; 4 Geisel School of Medicine at Dartmouth, Cancer Control Research Program, Norris Cotton Cancer Center, Lebanon, NH, USA; 5 University Medical Center Schleswig-Holstein, Institute for Medical Psychology and Medical Sociology, Kiel, Germany

**Keywords:** Physical activity, Cluster-randomized trial, Adolescents, School-based intervention, Pedometer

## Abstract

**Background:**

Physical activity during childhood and adolescence is associated with substantial health benefits and tracks into adulthood. Nevertheless, only 22.7% of German adolescents are sufficiently physically active. Thus, the promotion of an active lifestyle in youth is an essential issue of public health.

This study will evaluate the implementation and efficacy of the “läuft.” program to enhance physical activity in adolescence. “läuft.” is a multicomponent school-based program developed on the basis of effective strategies for health interventions and behavioral change.

**Methods/design:**

The “läuft.” physical activity program targets four different levels. (a) Each student receives a pedometer and documents his/her steps over 12 weeks using an interactive user account on the “läuft.” homepage. (b) For classes there will be different competitions, with achieving the most steps in selected weeks, the highest increases of steps and developing the most inventive ideas to promote physical activity in school. Besides, the intervention includes four educational lessons. (c) The headmasters and teaching staff of the participating schools will get information material with suggestions and encouragement to enhance physical activity in school. Participating teachers will be invited to an introductory seminar. (d) Parents will be provided with informational material about the program and will be invited to a parent-teacher conference about the benefits of being physically active and how they can support their children in engaging in a physically active lifestyle.

To evaluate the efficacy of the “läuft.” physical activity program, a two-arm cluster randomized controlled trial will be conducted in three waves: (1) baseline assessment, January/February 2014, (2) post assessment, June/July 2014 and (3) 12-month follow-up assessment, June/July 2015. Data collection will include physical and medical testing, self-administered questionnaires, group discussions and document analyses.

**Discussion:**

“läuft.” aims at fostering a physically active lifestyle in adolescence while a considerable decline of physical activity is present. Physical activity programs based in the school setting and following a multicomponent approach have been proven to be most successful. Furthermore, the use of pedometers is promising to enhance physical activity during the entire day and targets a wide range of adolescents regarding fitness and weight.

**Trial registration:**

Current Controlled Trials ISRCTN49482118.

## Background

There is extensive literature emphasizing physical activity as an important and modifiable factor influencing physical and mental health: being physically active on a regular basis is associated with substantial health benefits [1-4], including primary and secondary prevention of chronic diseases, a reduced risk of premature death and better mental health.

Physical activity in childhood and adolescence prevents a widespread variety of chronic diseases, including hypertension, obesity and depression [5,6]. An active lifestyle early in life is not just important for health outcomes in adolescence but may also be a significant factor for the level of activity in adulthood and health-related physical fitness [7-10]. Global recommendations on physical activity suggest that children and adolescents should take part in physical activity of moderate to vigorous intensity for at least 60 minutes per day to maintain health [11]. In consideration of epidemiological data, only 17.3% of female and 28.2% of male adolescents in Germany aged 11 to 17 years meet these recommendations [12]. Moreover, physical activity declines in older adolescents compared to children and younger adolescents [13]. Nader *et al*. reported a decline of moderate-to-vigorous physical activity from 180 minutes per day at 9 years to only 42 minutes per day at 15 years [14]. Hence, the promotion of an active lifestyle and physical fitness in early adolescence is an issue worthy of intervention development.

Considering the access to a large adolescent population, interventions based in the school setting are supposed to be the method of choice for increasing physical activity in youth [15]. Meta-analyses and reviews indicated positive impacts of school-based interventions for promoting physical activity on health-related indicators, namely an increase of rate and duration of in-school physical activity as well as aerobic fitness and a decrease of blood cholesterol and of physical inactivity [15-18]. Notably, multicomponent interventions involving peers, parents and communities were assumed to be highly effective [18]. However, despite these encouraging outcomes, almost no improvements in out-of-school physical activity has emerged from studies to date, and increases in activity level were rather short-term [17,18]. Therefore, school-based programs which aim to enhance physical activity and fitness in adolescents should also focus on changing the everyday routine to increase out-of-school physical activity and on establishing an active lifestyle in the long term.

There are several options for interventions to induce effects on physical activity throughout the entire day. One promising approach could be the use of pedometers which has already been shown to be effective in increasing physical activity among adolescents [19,20], not just in-school but also during out-of-school [21,22]. These findings are supplemented by studies emphasizing self-monitoring and goal-setting as one of the most effective intervention strategies to promote physical activity [23,24]. Both strategies can be easily and effectively combined with pedometer use [20]. The “läuft.” program integrates these strategies that have been proven to be successful in the promotion of physical activity into a multicomponent approach [15,18] that is school-based [15,17,18] and includes social support from peers and parents [25], goal-setting and self-monitoring through pedometer use [23,24].

### Objectives and hypotheses

The aim of the study is to evaluate the implementation and effectiveness of the “läuft.” physical activity program among adolescents in grade 8. Therefore, a two-arm three-wave cluster randomized controlled trial will be conducted. Expected effects on primary outcomes of the “läuft.” program in the intervention compared to the control group are defined as:

1. an increase in physical activity in general and in different contexts measured by questionnaires;

2. a decrease of sedentary behavior measured by questionnaires; and

3. an increase in cardiovascular fitness measured by the 20-meter shuttle run test.

In addition to primary outcomes, the following secondary outcomes will be included in the evaluation study of the “läuft.” physical activity program: body composition (that is, Body Mass Index (BMI), percentage of body fat mass and waist circumference), cardiovascular risk factors (that is, blood pressure, heart rate after stress as well as resting and recovery heart rate), mental health (that is, general self-efficacy, psychological well-being, self-esteem and depression), enjoyment of physical activity and sedentary behaviors, physical self-concept (that is, general physical capacity and physical attractiveness), self-efficacy towards physical activity, intention for physical activity and physical well-being.

## Methods/design

### Intervention

“läuft.” was designed by an interdisciplinary team of sport medicine specialists, sport scientists, teachers, nutritional scientists and psychologists.

**Figure 1:**
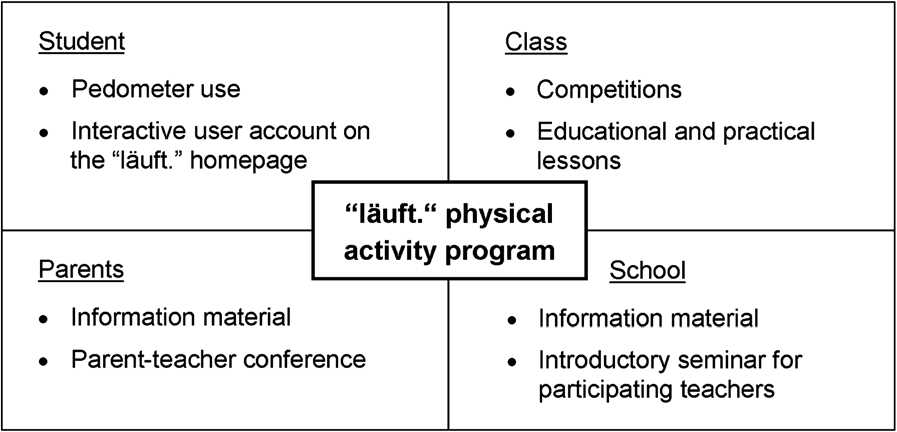
Overview of intervention components.

The intervention operates at four levels: individual, class, school and parents. The intervention period is 12 weeks. An overview of all components of the intervention is shown in Figure 1.

#### Class

The core components of the intervention are class competitions. On the one hand, each individual student in the class receives a pedometer and records the daily amount of steps over a period of 12 weeks. Steps are summed up to a class mean, documented on a class poster and reported to the project staff in three selected weeks. On the other hand, each class collects creative and inventive ideas on how to increase physical activity in everyday school life and keeps records of these ideas on the “läuft.” homepage (http://www.laeuft.info). In addition, the classes take part in four educational lessons: (1) introduction to the “läuft.” program, distribution of pedometers and informational material for students and their parents, (2) introduction to the creativity contest, (3) physical activity that combines running/walking with exercises at environmental facilities (for example, a bench, stairs or a jungle gym), and (4) reflection of strategies to take more steps in everyday life and setting of physical activity goals for the time period after the project. Besides, additional modules are offered that can be flexibly conducted during the program. The modules deal with topics such as the reflection of student’s physical activity, motives for being physically active or possible barriers to physical activity and how to handle them. Each lesson takes 45 minutes and will be conducted by teachers of the participating classes. After the end of the competition period, classes with the highest means of steps per week, with the largest increases and with the most inventive and creative class projects are awarded.

#### Individual

By using a pedometer, each student can evaluate his or her daily physical activity objectively and compare it to the class mean and common recommendations. Additionally, students can document their steps as well as their experiences during the program using a private and interactive user account on the “läuft.” homepage. Based on students’ entries, statistics (for example, mean of the week or day of best performance), a diagram to follow daily step totals over time and feedback will be delivered. Furthermore, the homepage provides opportunities to share experiences with other participating students and compete on step totals. The homepage is optimized for online use with smartphones.

#### School

The headmaster and entire teaching staff of each participating school will receive information material, including background and description of the program, activity-promoting teaching methods, suggestions on how to improve the school environment to stimulate physical activity, and related links leading to further information. All participating teachers will be offered an introductory seminar to learn more about the concept and components of the “läuft.” program.

#### Parents

A parent-teacher conference will take place at the class level to emphasize the importance of physical activity throughout the entire life and give parents ideas of how to support their children in establishing a physically active lifestyle. In addition, parents will receive information material about the program and the assessments. To take parents with a foreign background into account, materials will also be available in Turkish and Russian as the most frequently spoken foreign languages in Germany.

### Study design

The effectiveness of the “läuft.” physical activity program will be tested in a two-arm three-wave cluster randomized controlled trial. Data of the different waves are hypothesized to be clustered at the individual, class and school level. Schools are the units of randomization and will be randomly allocated to the intervention and control group. In intervention schools the “läuft.” physical activity program will be implemented, while schools of the control group run through the usual curriculum and will receive no further intervention. In both groups, fitness, medical and questionnaire data will be collected for three times: “baseline” (immediately before the intervention), “post” (immediately after the intervention) and “follow-up” (12 months after the end of the intervention) assessment. In addition, after the intervention, qualitative analyses will be conducted in classes of the intervention group to gain insight into interactive processes within the classes and to reconstruct subjective experiences and interpretations of activating-strategies.

**Figure 2:**
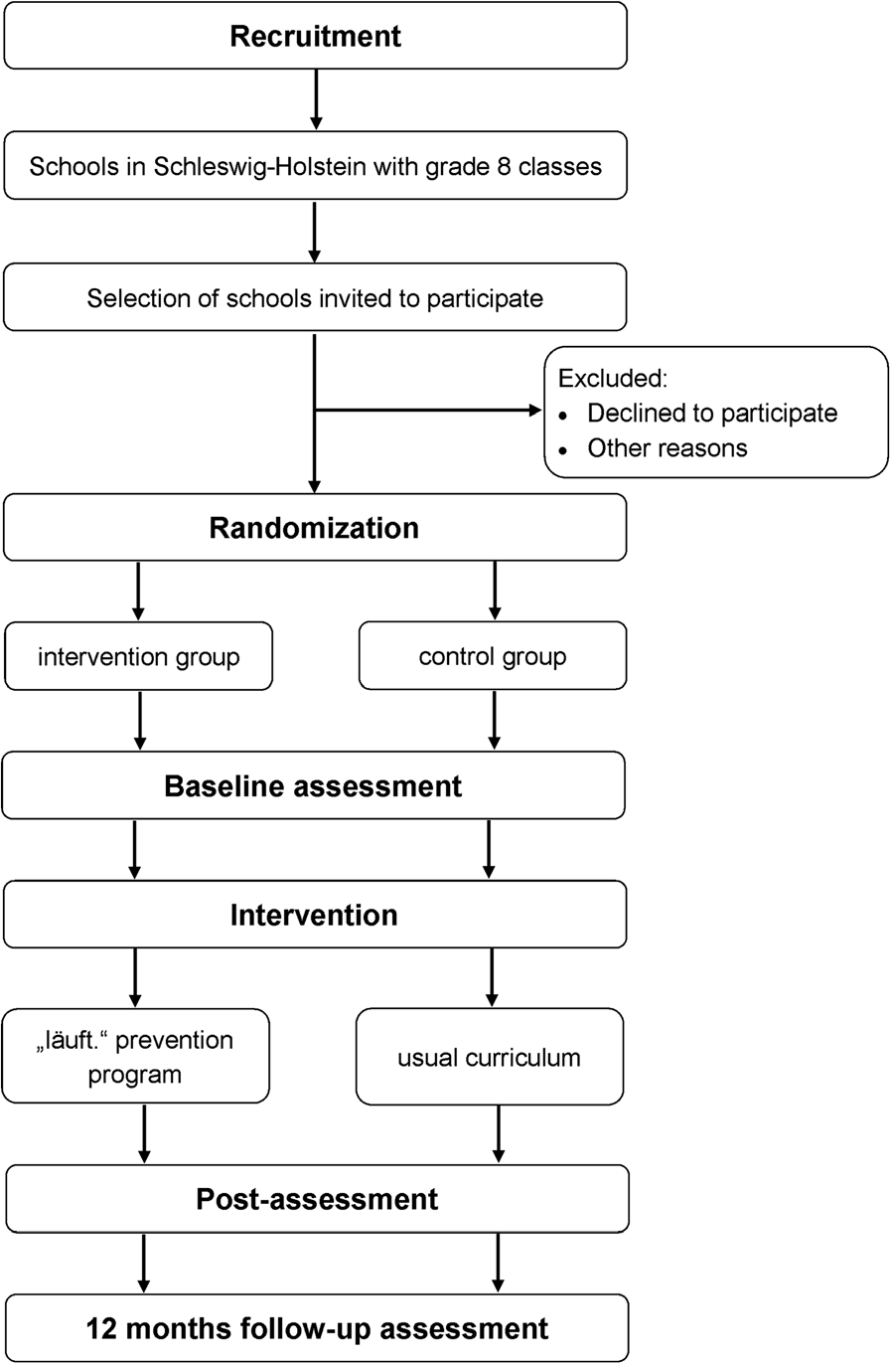
Study design.

Process evaluation of the planned trial will be realized through questionnaires and interviews during and after the study, respectively. The study design is presented in Figure 2.

### Sample size calculation

There are specific constraints for analysis of cluster randomized trials [26] since the responses of individuals within a cluster tend to be more similar than those of individuals of different clusters. This clustering effect can be defined as 1 + (m-1)p, where m is the average number of subjects per cluster and p the intraclass correlation coefficient (ICC) [27]. The ICC for physical fitness and adiposity was taken from another cluster randomized controlled trial, the Kindersportstudie (KISS) [28,29] and estimated with approximately 0.05 (p). Assuming an equal average cluster size of 20 pupils with parental consent (m = 20), a clustering effect of 1.95 was determined. Without taking into account the clustering of the data, assuming a small to medium effect size of the intervention (Cohen’s d = 0.30) on physical activity as one primary and the most important outcome being still detectable at follow-up, applying a significance level of alpha = 0.05 and power = 0.80, it can be estimated that N = 350 pupils are required to detect a difference between two independent sample means [30,31]. Taking the cluster effect into account, a required sample size of N = 683 students and 34 classes was determined. A computerized sample size calculator for cluster randomized trials was used to confirm this estimation [32].

Based on earlier experiences [33,34], a drop-out rate of 25% over the study was hypothesized. Hence, a total sample size of 911 pupils and 46 classes will be recruited for baseline to detect possible differences between the groups at follow-up. In order to recruit the estimated sample size of 46 classes while assuming a participation rate of 20% and an average of at least two participating classes per school, about 100 schools will be invited to participate.

### Procedure and participants

The study has been approved by the ethics committee of the German Psychological Society as well as the Ministry of Education and Science of Schleswig-Holstein. It targets 8^th^ grade classes with students aged 12 up to 15 years in secondary schools in the federal state of Schleswig-Holstein in Germany. Schools will be selected from a complete list of all secondary schools in Schleswig-Holstein obtained from the Ministry of Education. All types of schools will be included, except schools for disabled students. In order to recruit a study sample of 46 classes, about 100 schools will be invited to participate. An invitation letter and information sheets about the aims, methods and requirements of the study will be sent to the head of each school. Copies of the letters of approval by the Ministry of Education and the ethics committee will be attached. Schools agreeing to participate must send back their written consent indicating the acceptance of study characteristics (including randomization), number of grade 8 classes eligible for the study, names of the class teachers, and number of students per class. All students of participating classes will be included in the study. Schools not agreeing to participate are requested to explain their denial. In order to conduct non-responder analysis, all invited schools will be asked to provide some basic data about type and size of school, number of grade 8 classes, gym and sports equipment, sports facilities on the school ground and offered sports events, projects and courses. Participating schools will be stratified (type of school and number of grade 8 classes) and randomly allocated to the intervention and the control groups. Furthermore, parents will receive information sheets about the study and will be asked for informed consent to the storage and use of assessed data of their children. Teachers will register all names of students with no permission in a list which they keep throughout the entire trial. Students with refusals will take part in the assessment but their data will not be registered.

### Randomization

To avoid interference between the intervention and the control groups, stratified randomization will be carried out on the school level (according to the type of school and number of grade 8 classes). Expecting a higher drop-out of schools in the intervention group, randomization follows a 3:2 ratio, that is, each school will have a 3:2 chance of being assigned to the an intervention group. Randomization will be performed with a computer program.

### Data collection

From October 2013 to January 2014 the recruitment and randomization of schools will take place. In the intervention as well as in the control group, baseline data will be obtained in January and February 2014. The intervention program is scheduled for the period of March to May 2014. During this time the control group runs the usual curriculum. After 12 weeks, in the period of June to July 2014, post assessment data will be obtained. The follow-up assessment is planned for June and July 2015.

### Pilot study

The program has been tested, evaluated and adapted during a pilot study. From April to June 2013 a pilot study with a shorter intervention period (eight weeks) was conducted, including two schools with four classes and 89 students (41 female students; M_age_ = 13 years, SD = 0.50 years). To evaluate and improve the “läuft.” physical activity program, different qualitative and quantitative methods were employed: participant observation of the educational lessons, questionnaires for students and teachers to evaluate these lessons, and the assessment as well as interviews with students and teachers about the entire intervention. Based on the insights gained with respect to feasibility, comprehensibility and acceptance from the obtained data and experiences, the program was modified. The intervention was followed by one assessment in order to evaluate the feasibility of physical and medical testing as well as the comprehensibility of the questionnaire. The assessment was adapted in accordance with the experiences and results.

### Measures

The outcomes will be measured anonymously with self-administered questionnaires, physical testing and medical testing on the individual level as well as qualitative methods on the individual and the class level. An overview of all outcomes that will be assessed is presented in Table 1.

**Table 1:** Overview of measures

**Measurement methodology**	**Outcome**
**Medical testing**	Body weight and height (BMI percentiles)
	Body composition (fat mass)
	Waist circumference [35]
	Blood pressure
	Resting heart rate
	Heart rate after stress
	Recovery heart rate
**Physical testing**	Cardiovascular fitness [36]
**Questionnaire**	*Socio*-*demographic characteristics*
	Age
	Gender
	Migration background
	Socio-economic status [37]
	*Lifestyle Behavior*
	Physical activity* [38-40]
	Sedentary behavior [40]
	Use of tobacco [41]
	Use of alcohol [42,43]
	Food pattern [44]
	*Psychological constructs*
	General self-efficacy [45]
	Self-efficacy towards physical exercise [46]
	Self-concept for general physical capacity and physical attractiveness [47,48]
	Enjoyment of physical activity [49]
	Enjoyment of sedentary behavior [49]
	Sensation seeking [50]
	Physical well-being [51]
	Psychological well-being [51]
	Depression [52]
	Intention for physical activity [53]
	*Social factors*
	Parental support for physical activity [25]
	Friends physical activity*
	Normative expectations*
	Environmental situation to be physical active*
**Qualitative methods**	Activating-strategies (group discussion, document analysis)
	Interactive processes (group discussion)

*self-constructed items.

To permit a linking of individual information on subsequent assessments while assuring anonymity, each assessment-booklet will be labeled with a seven-digit individual code generated by the student [54].

#### Medical testing

Standing height will be determined using a portable stadiometer (Seca 213, Basel, Switzerland) and body weight as well as body composition (fat mass) using bioelectrical impedance analysis (Omron BF-511, Omron Healthcare, Mannheim, Germany). Students’ BMI will be calculated and converted into age- and gender-specific percentiles using the Coles LMS-method [55] on the basis of a representative German sample [56]. For further analysis, the standard deviation scores will be determined. Blood pressure and resting heart rate will be measured with an automated oscillometric device (M5-Professional, Omron Healthcare, Mannheim, Germany). A flexible tape will be used to determine waist circumference at the medial axillary line half-way between the lowest rib and the iliac crest [35]. For measuring heart rate after stress as well as recovery heart rate after three minutes, respectively, pulse watches with chest belts will be used (Polar FT1, Polar Electro GmbH, Büttelborn, Germany). The entire medical testing will be conducted under a physician’s supervision.

#### Physical testing

For measuring cardiovascular fitness, the 20-meter shuttle run test will be conducted [36]. In this progressive running test, the students have to run between two lines for a distance of 20 meters. The required running pace of an initial 8.0 km/h increases continuously by 0.5 km/h each minute. Students have to cross the opposite line in a given time interval indicated by prerecorded audio signals. The test stops when the student fails to reach the line by the sound for the second time. To determine aerobic capacity, the number of one-minute stages will be counted with a precision of 0.5 stages. The 20-meter shuttle run test allows a validated estimation of maximal oxygen uptake as an indicator of cardiovascular fitness [57,58].

#### Questionnaire

Data about socio-demographic characteristics, lifestyle behaviors well as psychological and social variables will be collected by self-administered questionnaires. The items and scales used are based on published and validated questionnaires and the international literature. The entire questionnaire was tested for feasibility and comprehensibility during the pilot study with 89 students. Based on the experiences in the pilot study regarding comprehensibility and acceptance of items and conducted item analyses, the questionnaire has been revised and shortened. Considering item analyses, items have been excluded if (a) item difficulty was smaller than 20% and larger than 80%, respectively, (b) item-rest correlation was smaller than .40 and (c) their exclusion either led to higher internal consistencies or did not substantially decrease internal consistency of the particular scale.

Table 2 summarizes item characteristics and internal consistencies of the scales that have been modified based on the results of the pilot study and that will be employed in the planned study. Furthermore, for self-constructed items and scales seven-day retest-reliabilities were determined on a subsample of 38 students.

**Table 2:** Item characteristics and internal consistencies of modified scales in their final version

		**Pilot study (N = 89)**		
**Scales**	**Number of Items**	**Cronbach’s α**	**Item**-**rest correlation**	**Item difficulty (in%)**
General self-efficacy	5	.83	.58 to .66	60.8 to 68.2
Self-concept: general physical capacity	5	.92	.78 to .83	50.6 to 70.5
Self-concept: physical attractiveness	5	.84	.58 to .71	57.9 to 76.1
Sensation seeking	2	.86		41.0, 43.6
Self-efficacy for physical activity	8	.87	.56 to .78	42.9 to 70.5
Physical activity enjoyment	10	.92	.58 to .85	53.9 to 75.6
Sedentary behavior enjoyment (screen-based)*	4			
Sedentary behavior enjoyment (non-screen-based)	4	.89	.57 to .84	54.2 to 66.4
Parental support for physical activity	2	.79		31.3, 34.8
Psychological well-being	5	.80	.46 to .72	55.3 to 74.4
Physical well-being	2	.70		59.8, 66.1

*In the pilot questionnaire there were two separated scales for TV/DVD time and computer use. Both scales will be combined as overall screen-based sedentary behavior. Hence, there are no item characteristics available for this combined scale.

##### Socio-demographic characteristics

Socio-demographic characteristics like school type, age, gender and migration background will be assessed as well as the socio-economic status asking for the parents’ highest educational attainment [37].

##### Lifestyle behavior

Adapted items of the MoMo-activity-questionnaire [38] will be used to assess physical activity in different contexts (school, sports clubs and leisure time) and in different dimensions (duration, frequency and intensity) as well as the mode of commuting to school. These questions provide information about physical activity during a regular week. Besides, students in the pilot study were asked with which activities they choose to spend their recess time in school. The three activities that were named the most (sitting or standing while talking to others, walking through the school or schoolyard, and sports such as football or basketball) will be included in the final questionnaire. Students will be asked how often they engaged in these activities during the recess times of the last week. Overall moderate to vigorous physical activity will be determined by a reliable and validated two-item screening measure [39]. The survey method for leisure time sedentary behavior was modified from Zabinski and colleagues [40]. Students will be asked how much time they spent on the most recent school day and the most recent Sunday with different sedentary behaviors. Different physical activities were added to the list providing insights into the distribution of sedentary and active leisure time during a specific day. The list of activities is based on the original questionnaire, categories for physical activity (active transport, household activities, physical activity in sports clubs, in school and in leisure time), and extended by answers of students in the pilot study. Lifetime smoking and current smoking [41] as well as lifetime alcohol use, current alcohol use and frequency/number of previous binge drinking [42,43] will be assessed. Finally, students will be asked how often they consume different foodstuff in a usual week, namely, fresh fruits and vegetables, sweets or chocolate, soft drinks, fast food and water [44] (retest-reliability (r) ranged from 0.39 to 0.63).

##### Psychological variables

Items of published and validated scales selected with respect to item analysis during the pilot study will be used for the assessment of general self-efficacy [45], self-efficacy towards physical activity [46], physical self-concept for general physical capacity and physical attractiveness [47,48], enjoyment of physical activity PACES (Physical Activity Enjoyment Scale) [49], sensation seeking [50] as well as physical and psychological well-being [51]. The enjoyment of screen-based and non-screen-based sedentary behaviors will be assessed with four adapted items of the PACES [49] (non-screen-based sedentary behavior: r = .59). The scale “depressed affect” of the German version of the Center for Epidemiological Studies Depression Scale for Children CES-DC [52,59] and three items to assess the intention for physical activity were added to the questionnaire after the pilot study. Thus, students will be asked how much they agree with three given statements, (for example: “I plan to be physically active for at least 60 minutes per day on most days of the next week.”; seven-point semantic differential scale from “likely” to “unlikely”) [53].

##### Social variables

Parental support for physical activity will be assessed by an adapted version of a scale used by Trost *et al*. [25] (r = .80). Furthermore, students will be asked how many of their friends are physically active regularly (5-point scale: “none” to “all”, r = .72), how many are in a sports’ club (5-point scale: “none” to “all”, r = .69), how important it is in their circle of friends to be physically active (4-point scale: “not important” to “very important”, r = .50) and how often their parents were physically active without them in the past week (5-point scale: “never” to “more than five times”, r = .78). Two items will be used to obtain information about the environmental situation to be physically active: “In my neighborhood there are many facilities in which for being physically active.” (5-point scale: “strongly disagree” to “strongly agree”, r = .60) and “When I want to do sports, facilities and equipment are lacking.” (5-point scale: “never” to “always”, r = .70). Finally, normative expectations of physical activity and screen time will be assessed. Therefore, students will be asked for their estimation of the portion of adolescents that are physically active for 60 minutes on a daily basis (r = .60) and how many hours per day adolescents spend watching TV or on the computer (r = .61).

#### Qualitative methods

In addition to and in combination with quantitative methods, a qualitative analysis of processes and outcomes of the “läuft.” program will be performed. During the program, document analyses of students’ entries on the homepage will be conducted. Group discussions with students will be carried out in the course of the post-assessment.

##### Document analysis

To gain insights into processes of the intervention immediately, a qualitative document analysis of activities on the “läuft.” homepage will be conducted. The homepage can be used by students to take part in the creativity contest, compete with their amount of steps, share their individual experiences in the program, discuss program associated topics and participate in weekly tasks. Students’ activities and entries on the homepage will provide information about the activating-strategies that will be used and developed during the intervention.

##### Group discussion (students’ perspective)

In order to obtain a deeper understanding of students’ experiences, motives and interpretations of given impulses and activating-strategies during the program, group discussions with selected members of each intervention class will be conducted in the course of the post-assessment. On the basis of a theoretical sampling [60], five to seven students differing with regard to (a) gender, (b) interest in and commitment to the “läuft.” program and (c) athleticism will be asked to participate in the group discussions. These will follow a thematic compendium and will be carried out in accordance with the principles of Bohnsack [61], which emphasize the self-deployment of the group and the minor impact of the researcher on group processes.

### Process evaluation

To evaluate the quality of implementation, teachers will be asked to fill in questionnaires about the realization of each intervention component during the program. Information about acceptance, feasibility and suggestions for improving the program will be provided by another questionnaire for all participating teachers after the intervention and interviews with selected teachers. The latter will additionally enable a more profound and case-oriented analysis of interaction processes, barriers and success strategies. The teachers will be selected using theoretical sampling based on results of the questionnaires as well as formal criteria (for example, gender and type of school).

### Statistical analysis

Statistical analysis will be conducted with Stata V13.1 (Stata Corp, College Station, TX, USA) following the intention to treat principle. To evaluate efficacy of the program, multilevel analysis with the levels: schools, classes, individuals and waves, will be performed. Random intercepts will be included for schools, classes and individuals taking the clustering effect into account. Group and covariates will be taken as fixed effects. Differences between intervention and control group at baseline as well as attrition analysis will be examined using a t-test for independent samples and Fisher’s exact test.

### Qualitative analysis

Qualitative data will be analyzed using ATLAS.ti software (ATLAS.ti GmbH, Berlin, Germany). Following basic methodological principles of the qualitative content analysis [62] and coding strategies of the grounded theory approach [63], relevant categories will be generated, systemized and summarized to permit thorough interpretations of participants’ opinions and experiences.

## Discussion

The described study protocol is intended to evaluate the efficacy of the “läuft.” physical activity program to enhance physical activity of adolescents using a two-arm three-wave cluster randomized controlled trial. “läuft.” is a school-based program addressing an age group - adolescents aged 13 to 15 years - in which the level of physical activity declines dramatically [13,14]. Thus, to accomplish an increase of the basic physical activity level in everyday life the intervention is targeted on four different components: individual, class, school and parents.

In the promotion of physical activity in adolescence, a multicomponent approach and interventions in the school setting have been proven to be most promising and successful strategies [15]. Considering the easy access to students from all levels of society and the long period of time they spend in school, health programs in the school setting can effectively reach a large number of adolescents. On the other hand, many school-based interventions were insufficient in enhancing not just in-school but also out-of-school physical activity [17,18]. The “läuft.” physical activity program tries to overcome this shortcoming by changing students’ everyday routine by using pedometers throughout the entire day. Moreover, pedometers have been proven to increase physical activity also in low-active adolescents [21,22] who are at high risk of adiposity, and poorer cardiovascular health and fitness [64]. The implementation of a follow-up assessment 12 months after the program ends, takes into account that interventions for promoting physical activity in adolescents are mostly limited to rather short-term effects [17]. Considering four educational lessons over a period of 12 weeks, time and effort for participating teachers are rather low. Indeed, the intervention might not be intensive enough to lead to major improvements in cardiovascular risk factors, such as BMI or blood pressure, but the low threshold for successful participation might also cause better acceptance. Students do not need to be athletic or of normal weight to achieve a high number of steps.

Nevertheless, universally applied programs also include students that are already sufficiently physically active. Thus, not all resources will be used efficiently. As the main limitation of the presented study, physical activity will be assessed by using self-administered questionnaires and will not be measured objectively through accelerometers. Even though, with respect to adolescents, reliability and validity of self-administered recall-questionnaires are acceptable to good [65]; aiming for triangulation of quantitative and qualitative research methods, the qualitative results pose an opportunity to reach a better and deeper understanding of the barriers and prospects of a “successful” intervention and aspired sustainability. Suitable strategies and options can be deducted from the quantitative-qualitative data and lead to concrete recommendations for future implementation.

## Trial status

At the time of manuscript submission (September 2013), the school recruitment was about to begin and will finally be started in October 2013. First data assessment will start in 2014.

## Abbreviations

BMI: Body mass index; CES-DC: Center for epidemiological studies depression scale for children; ICC: Intraclass correlation coefficient; PACES: Physical activity enjoyment scale; r: Retest-reliability.

## Competing interests

The authors declare that they have no competing interests.

## Authors’ contributions

RH is the principal investigator. RH and BI supervise the study. RH, BI, JH, CK, BW and JS took part in the study concept and design. RH, BI, JH, MJ, KM, VS, IS and CK were responsible for the intervention concept. IS, MJ and BW were responsible for determination of the medical assessment procedure. MJ and IS were responsible for the determination of physical testing. VS, BI and RH constructed the questionnaire. KM and CK determined the qualitative methods. MJ, VS, KM, IS, BI and CK acquired the data during the pilot study. VS and BI analyzed the statistical data of the pilot study. KM and CK analyzed the qualitative data of the pilot study. VS, KM, MJ, IS, BI, CK and RH interpreted the data of the pilot study. VS and JH drafted the manuscript. RH, BI, MJ, KM, CK, IS, BW and JS performed the critical revision of the manuscript for important intellectual content. All authors read and approved the final manuscript.

## References

[B1] World Cancer Research Fund / American Institute for Cancer Research Food, Nutrition, Physical Activity, and the Prevention of Cancer: a Global Perspective 2007 Washington, DC: AICR

[B2] Warburton DE Nicol CW Bredin SS Health benefits of physical activity: the evidence CMAJ 2006 174 801 809 10.1503/cmaj.051351 16534088 PMC1402378

[B3] US Department of Health and Human Services Physical Activity and Health: A Report of the Surgeon General 1996 Atlanta, GA: U.S: Department of Health and Human Services, Centers for Disease Control and Prevention, National Center for Chronic Disease Prevention and Health Promotion

[B4] Lee IM Shiroma EJ Lobelo F Puska P Blair SN Katzmarzyk PT Effect of physical inactivity on major non-communicable diseases worldwide: an analysis of burden of disease and life expectancy Lancet 2012 380 219 229 10.1016/S0140-6736(12)61031-9 22818936 PMC3645500

[B5] Janssen I LeBlanc AG Systematic review of the health benefits of physical activity and fitness in school-aged children and youth Int J Behav Nutr Phys Act 2010 7 40 10.1186/1479-5868-7-40 20459784 PMC2885312

[B6] Ekelund U Luan J Sherar LB Esliger DW Griew P Cooper A Moderate to vigorous physical activity and sedentary time and cardiometabolic risk factors in children and adolescents JAMA 2012 307 704 712 10.1001/jama.2012.156 22337681 PMC3793121

[B7] Malina RM Physical activity and fitness: pathways from childhood to adulthood Am J Hum Biol 2001 13 162 172 10.1002/1520-6300(200102/03)13:2<162::AID-AJHB1025>3.0.CO;2-T 11460860

[B8] Telama R Tracking of physical activity from childhood to adulthood: a review Obes Facts 2009 2 187 195 10.1159/000222244 20054224 PMC6516203

[B9] Hasselstrom H Hansen SE Froberg K Andersen LB Physical fitness and physical activity during adolescence as predictors of cardiovascular disease risk in young adulthood: Danish youth and sports study: an eight-year follow-up study Int J Sports Med 2002 23 S27 S31 10.1055/s-2002-28458 12012259

[B10] Twisk JW Kemper HC van Mechelen W The relationship between physical fitness and physical activity during adolescence and cardiovascular disease risk factors at adult age. The Amsterdam Growth and Health Longitudinal Study Int J Sports Med 2002 23 Suppl 1 S8 S14 12012256 10.1055/s-2002-28455

[B11] World Health Organization Global Recommendations on Physical Activity for Health 2010 Geneva: World Health Organization 26180873

[B12] Lampert T Mensink GB Romahn N Woll A Physical activity among children and adolescents in Germany. Results of the German Health Interview and Examination Survey for Children and Adolescents (KiGGS). Bundesgesundhbl Gesundheitsforsch Gesundheitsschutz 2007 50 634 642 10.1007/s00103-007-0224-8 17514447

[B13] Dumith SC Gigante DP Domingues MR Kohl HW 3rd Physical activity change during adolescence: a systematic review and a pooled analysis Int J Epidemiol 2011 40 685 698 10.1093/ije/dyq272 21245072

[B14] Nader PR Bradley RH Houts RM McRitchie SL O’Brien M Moderate-to-vigorous physical activity from ages 9 to 15 years JAMA 2008 300 295 305 10.1001/jama.300.3.295 18632544

[B15] Kriemler S Meyer U Martin E van Sluijs EM Andersen LB Martin BW Effect of school-based interventions on physical activity and fitness in children and adolescents: a review of reviews and systematic update Br J Sports Med 2011 45 923 930 10.1136/bjsports-2011-090186 21836176 PMC3841814

[B16] Dobbins M De Corby K Robeson P Husson H Tirilis D School-based physical activity programs for promoting physical activity and fitness in children and adolescents aged 6–18 Cochrane Database Syst Rev 2009 1 CD007651 10.1002/14651858.CD007651 19160341

[B17] De Meester F van Lenthe FJ Spittaels H Lien N de Bourdeaudhuij I Interventions for promoting physical activity among European teenagers: a systematic review Int J Behav Nutr Phys Act 2009 6 6 10.1186/1479-5868-6-6 19961623 PMC2795736

[B18] van Sluijs EM McMinn AM Griffin SJ Effectiveness of interventions to promote physical activity in children and adolescents: systematic review of controlled trials Br J Sports Med 2008 42 653 657 18685076

[B19] Kang M Marshall SJ Barreira TV Lee JO Effect of pedometer-based physical activity interventions: a meta-analysis Res Q Exerc Sport 2009 80 648 655 19791652 10.1080/02701367.2009.10599604

[B20] Lubans DR Morgan PJ Tudor-Locke C A systematic review of studies using pedometers to promote physical activity among youth Prev Med 2009 48 307 315 10.1016/j.ypmed.2009.02.014 19249328

[B21] Schofield L Mummery WK Schofield G Effects of a controlled pedometer-intervention trial for low-active adolescent girls Med Sci Sports Exerc 2005 37 1414 1420 10.1249/01.mss.0000174889.89600.e3 16118591

[B22] Lubans D Morgan P Evaluation of an extra-curricular school sport programme promoting lifestyle and lifetime activity for adolescents J Sports Sci 2008 26 519 529 10.1080/02640410701624549 18274949

[B23] Michie S Abraham C Whittington C McAteer J Gupta S Effective techniques in healthy eating and physical activity interventions: a meta-regression Health Psychol 2009 28 690 701 19916637 10.1037/a0016136

[B24] Ory MG Jordan PJ Bazzarre T The behavior change consortium: setting the stage for a new century of health behavior-change research Health Educ Res 2002 17 500 511 10.1093/her/17.5.500 12408195

[B25] Trost SG Sallis JF Pate RR Freedson PS Taylor WC Dowda M Evaluating a model of parental influence on youth physical activity Am J Prev Med 2003 25 277 282 10.1016/S0749-3797(03)00217-4 14580627

[B26] Campbell MJ Donner A Klar N Developments in cluster randomized trials and statistics in medicine Stat Med 2007 26 2 19 10.1002/sim.2731 17136746

[B27] Murray DM Varnell SP Blitstein JL Design and analysis of group-randomized trials: a review of recent methodological developments Am J Public Health 2004 94 423 432 10.2105/AJPH.94.3.423 14998806 PMC1448268

[B28] Zahner L Puder JJ Roth R Schmid M Guldimann R Pühse U Knöpfli M Braun-Fahrländer C Marti B Kriemler S A school-based physical activity program to improve health and fitness in children aged 6–13 years (“Kinder-Sportstudie KISS”): study design of a randomized controlled trial [ISRCTN15360785] BMC Public Health 2006 6 147 10.1186/1471-2458-6-147 16756652 PMC1513202

[B29] Kriemler S Zahner L Schindler C Meyer U Hartmann T Hebestreit H Brunner-La Rocca HP van Mechelen W Puder JJ Effect of school based physical activity programme (KISS) on fitness and adiposity in primary schoolchildren: cluster randomised controlled trial BMJ 2010 340 c785 10.1136/bmj.c785 20179126 PMC2827713

[B30] Cohen J Statistical Power Analyses for Behavioral Science 1988 Hilldale, New York: Erlbaum

[B31] Cohen J A power primer Psychol Bull 1992 112 155 159 19565683 10.1037//0033-2909.112.1.155

[B32] Campbell MK Thomson S Ramsay CR MacLennan GS Grimshaw JM Sample size calculator for cluster randomized trials Comput Biol Med 2004 34 113 125 10.1016/S0010-4825(03)00039-8 14972631

[B33] Hanewinkel R Sargent JD Longitudinal study of exposure to entertainment media and alcohol use among German adolescents Pediatrics 2009 123 989 995 10.1542/peds.2008-1465 19255030 PMC2746499

[B34] Hanewinkel R Isensee B Sargent JD Morgenstern M Cigarette advertising and adolescent smoking Am J Prev Med 2010 38 359 366 10.1016/j.amepre.2009.12.036 20307803

[B35] World Health Organisation Obesity: Preventing and Managing the Global Epidemic 2004 Geneva: World Health Organization 11234459

[B36] Leger LA Mercier D Gadoury C Lambert J The multistage 20 metre shuttle run test for aerobic fitness J Sports Sci 1988 6 93 101 10.1080/02640418808729800 3184250

[B37] Kunter M Schümer G Artelt C Baumert J Klieme E Neubrand M Prenzel M Schiefele U Schneider W Stanat P Tillmann K-J Weiß M Pisa 2000: Documentation of measures 2002 Berlin: Max-Planck-Institut für Bildungsforschung

[B38] Bös K Worth A Heel J Opper E Romahn N Tittelbach S Wank V Woll A Testmanual des Motorik Moduls im Rahmen des Kinder- und Jugendgesundheitssurveys des Robert-Koch-Instituts Haltung Bewegung 2004 24 3 41

[B39] Prochaska JJ Sallis JF Long B A physical activity screening measure for use with adolescents in primary care Arch Pediatr Adolesc Med 2001 155 554 559 10.1001/archpedi.155.5.554 11343497

[B40] Zabinski MF Norman GJ Sallis JF Calfas KJ Patrick K Patterns of sedentary behavior among adolescents Health Psychol 2007 26 113 120 17209704 10.1037/0278-6133.26.1.113

[B41] World Health Organization Guidelines for Controlling and Monitoring the Tobacco Epidemic 1998 Geneva: World Health Organization

[B42] Lintonen T Ahlström S Metso L The reliability of self-reported drinking in adolescence Alcohol Alcohol 2004 39 362 368 10.1093/alcalc/agh071 15208172

[B43] Wechsler H Nelson TF Binge drinking and the American college student: what’s five drinks? Psychol Addict Behav 2001 15 287 291 11767258 10.1037//0893-164x.15.4.287

[B44] Ravens-Sieberer U Thomas C Gesundheitsverhalten von Schülern in Berlin: Ergebnisse der HBSC-Jugendgesundheitsstudie 2002 im Auftrag der WHO 2003 Berlin: Robert Koch Institut

[B45] Schwarzer R Jerusalem M Skalen zur Erfassung von Lehrer- und Schülermerkmalen. Dokumentation der psychometrischen Verfahren im Rahmen der Wissenschaftlichen Begleitung des Modellversuchs Selbstwirksame Schulen 1999 Berlin: Freie Universität Berlin

[B46] Fuchs R Schwarzer R Self-efficacy towards physical exercise: reliability and validity of a new instrument Z Different Diagnost Psychol 1994 15 141 154

[B47] Stiller J Würth S Alfermann D The measurement of physical self-concept (PSK) - the development of the PSK-scales for children, adolescents, and young adults Z Different Diagnost Psychol 2004 25 239 257 10.1024/0170-1789.25.4.239

[B48] Brettschneider AK Rosario AS Ellert U Validity and predictors of BMI derived from self-reported height and weight among 11- to 17-year-old German adolescents from the KiGGS study BMC Res Notes 2011 4 414 10.1186/1756-0500-4-414 22005143 PMC3216908

[B49] Jekauc D Voelkle M Wagner MO Mewes N Woll A Reliability, validity, and measurement invariance of the German version of the physical activity enjoyment scale J Pediatr Psychol 2013 38 104 115 10.1093/jpepsy/jss088 22946084

[B50] Stephenson MT Hoyle RH Palmgreen P Slater MD Brief measures of sensation seeking for screening and large-scale surveys Drug Alcohol Depend 2003 72 279 286 10.1016/j.drugalcdep.2003.08.003 14643945

[B51] Erhart M Ellert U Kurth BM Ravens-Sieberer U Measuring adolescents’ HRQoL via self reports and parent proxy reports: an evaluation of the psychometric properties of both versions of the KINDL-R instrument Health Qual Life Outcomes 2009 7 77 10.1186/1477-7525-7-77 19709410 PMC2749015

[B52] Faulstich ME Carey MP Ruggiero L Enyart P Gresham F Assessment of depression in childhood and adolescence: an evaluation of the center for epidemiological studies depression scale for children (CES-DC) Am J Psychiatry 1986 143 1024 1027 3728717 10.1176/ajp.143.8.1024

[B53] Hagger MS Chatzisarantis N Biddle SJ Orbell S Antecedents of children’s physical activity intentions and behaviour: predictive validity and longitudinal effects Psychol Health 2001 16 391 407 10.1080/08870440108405515

[B54] Galanti MR Siliquini R Cuomo L Melero JC Panella M Faggiano F Testing anonymous link procedures for follow-up of adolescents in a school-based trial: The EU-DAP pilot study Prev Med 2007 44 174 177 10.1016/j.ypmed.2006.07.019 16979751

[B55] Cole TJ The LMS method for constructing normalized growth standards Eur J Clin Nutr 1990 44 45 60 2354692

[B56] Kromeyer-Hauschild K Wabitsch M Kunze P Geller F Geiß HC von Hippel A Johnsen D Korte W Müller G Müller JM Niemann-Pilatus A Remer T Schaefer F Wittchen H-U Zabransky S Zellner K Ziegler A Hebebrand J Percentiles of body mass index in children and adolescents evaluated from different regional German studies Monatsschr nderheilkd 2001 149 807 818

[B57] Mahar MT Guerieri AM Hanna MS Kemble CD Estimation of aerobic fitness from 20-m multistage shuttle run test performance Am J Prev Med 2011 41 S117 S123 10.1016/j.amepre.2011.07.008 21961611

[B58] van Mechelen W Hlobil H Kemper HC Validation of two running tests as estimates of maximal aerobic power in children Eur J Appl Physiol Occup Physiol 1986 55 503 506 10.1007/BF00421645 3769907

[B59] Barkmann C Erhart M Schulte-Markwort M The German version of the Centre for epidemiological studies depression scale for children: psychometric evaluation in a population-based survey of 7 to 17 years old children and adolescents–results of the BELLA study Eur Child Adolesc Psychiatry 2008 17 Suppl 1 116 124 19132311 10.1007/s00787-008-1013-0

[B60] Hammersley M Jupp V Theoretical sampling The Sage Dictionary of Social Research Methods 2006 London: SAGE Publications 298 299

[B61] Bohnsack R Flick U, von Kardorff E, Steinke I Group discussion Qualitative Research - A Handbook 2003 Reinbek: Rowohlt Taschenbuch Verlag 369 384

[B62] Mayring P Qualitative Content Analysis. Basics and Methods 2000 Beltz: Weinheim, Basel

[B63] Glaser B Strauss A The Discovery of Grounded Theory: Strategies for Qualitative Research 1967 New York: Aldine de Gruyter

[B64] Strong WB Malina RM Blimkie CJ Daniels SR Dishman RK Gutin B Hergenroeder AC Must A Nixon PA Pivarnik JM Rowland T Trost S Trudeau F Evidence based physical activity for school-age youth J Pediatr 2005 146 732 737 10.1016/j.jpeds.2005.01.055 15973308

[B65] Trost SG State of the art reviews: measurement of physical activity in children and adolescents Am J Lifestyle Med 2007 1 299 314 10.1177/1559827607301686

